# Granular cell tumor of the vulva: Case report and systematic review of the literature

**DOI:** 10.1097/MD.0000000000032568

**Published:** 2022-12-30

**Authors:** Guang-Yao Lin, Yan Liu, Tao Ye, Xin-Yu Lu, Jie Gao, Yong-Zhou Wang

**Affiliations:** a Department of Gynecology, Longhua Hospital, Shanghai University of Traditional Chinese Medicine, Shanghai, China; b Department of Gynecology, The Affiliated Traditional Chinese Medicine Hospital of Southwest Medical University, Luzhou, China; c Department of Radiology, The Affiliated Traditional Chinese Medicine Hospital of Southwest Medical University, Luzhou, China; d Department of Pathology, The Affiliated Traditional Chinese Medicine Hospital of Southwest Medical University, Luzhou, China.

**Keywords:** case report, granular cell tumor, systematic review, the vulva, Z-plasty

## Abstract

Granular cell tumor (GCT) of the vulva is an exceptionally rare female genital tract tumor. The majority of these are benign and there are no standardized surgical techniques for the special site to reduce tension of the wound.

**Patient concerns:**

A 47-years-old Chinese woman experienced a nodule on her right vulva with itch sometimes in late 2018.

**Diagnoses:**

Magnetic resonance imaging showed a high possibility of vulvar cancer. While Chest X-ray, abdominal sonography, and cystoscopy examination were unremarkable.

**Interventions:**

The patient underwent local complete resection of vulvar tumor under general anesthesia on March 24, 2022. The resection scope was approximately 4 cm × 3 cm × 3 cm. Due to the large surgical incision, Z-plasty was performed to achieve the primary closure for decreasing wound tension and improving aesthetic reduction.

**Outcomes:**

The final pathological diagnosis was benign GCT of the vulva and surgical margins were uninvolved. At 8 months follow-up, no new lesions were detected.

**Lessons:**

Surgery with negative resection margins is the mainstay for benign GCT of the vulva, while Z-plasty is appropriate for decreasing the tension of the wound and improving aesthetic reduction.

## 1. Introduction

Granular cell tumor (GCT) is a soft tissue tumor consisting of eosinophilic granular cytoplasm, which can be found throughout the body. This tumor typically affects in the skin and subcutaneous tissues, the breast, the head and neck.^[1]^ For example, in a Veterans Administration Hospital, Jobrack et al viewed 13 cases over a period of 20 years and found 8 cases were in the skin and subcutaneous tissue.^[2]^ Moreover, Curtis et al identified 18 cases aged from 23 to 76 years with an incidence of 38.8% and 16.6% located on the tongue and lip, respectively.^[1]^ In contrast, granular cell tumor of the vulva is much rarer among women of various ages. The clinical presentation of GCT of the vulva is diverse. Tumors are often described as slowly growing, skin color, small, pain, slightly itchy and may also appear as firm and non-tender nodules.^[3–6]^ GCT of the vulva often presents as solitary or multiple, distinct lesions. Therefore, a thorough physical examination is needed in order not to overlook a nodule without any symptoms. Diagnostic delay is another vital issue which, to some extent, could neglect malignant GCT of the vulva.^[7]^ Because many patients reported in the literature, plenty of time delay between the onset of the nodule and the final histological diagnosis of GCT of the vulva, has been noted.^[6]^

Clearly, due to the rarity of GCTs, it is difficult to diagnose and distinguish with vulvar sebaceous cyst. In contrast, there is no consensus on the diagnostic criteria for women with this disease. Based on a large series of 73 cases with GCTs, in 1998s, Fanburg-Smith et al proposed 6 histologic criteria: necrosis, spindling, vesicular nuclei with large nucleoli, increased mitotic activity (>2 mitoses in 10 high-power fields at 200x fields), high nuclear to cytoplasmic ratio, and pleomorphism.^[8]^ Malignant GCTs met 3 or more of these criteria; benign GCTs revealed only focal pleomorphism but satisfied none of the criteria, and atypical GCTs met 1 or 2 criteria. By classifying 48 cases according to the criteria above, in 2011s, Nasser et al found using necrosis and/or mitoses alone could reclassify 37 benign cases into 44 and all of them did not recur or metastasize.^[9]^ Significantly, histopathological characteristics of local invasion are not associated with adverse prognosis. For example, in a series of 119 women with GCTs from a multicenter of France, Battistella et al reported vascular invasion occurred in 27 (23%) cases consisting of subendothelial layers infiltration. During available follow-up (range: 2 to 24 months), no metastases, no local recurrence, and no deaths were documented which were conflict with the 6 classical criteria above.^[10]^ Besides, computed tomography, magnetic resonance imaging (MRI), and ^18^F-FDG PET/computed tomography are beneficial in assessing the size of the tumor and its association with the surrounding tissue or metastases.^[11]^ These data above also suggest that it may be challenging to diagnose GCTs.

The most used treatment specimens for GCT of the vulva were excision. Despite, this disease is most often benign, its potential aggressive behavior may cause serious complications if it occurs in critical locations. To reduce the incidence of recurrence and overlook malignant ones, wide local excision with negative margins is necessary.^[12]^ In contrast to 20% recurrence rates with positive margins, the clear margins are 2% to 8%.^[13]^ However, once diagnosed with malignant GCT histologically, wide local excision combined with regional lymph node dissection or radiotherapy for widespread regional metastasis are suggested.^[7,14]^ Typically, the metastases within 2 years are reported in the majority of malignant cases, and the rate of mortality is approximately close to 60 % within 3 years.^[15]^

To highlight the clinical characteristics, diagnosis, and management of women with GCT of the vulva, we report a rare case of a 47-years-old woman with histologically verified GCT of the vulva. In addition, a systematic review on the literature of similar case reports of women with GCT of the vulva, the challenge of diagnosis, and the most common therapies are discussed.

## 2. Case presentation

**Figure 1.**
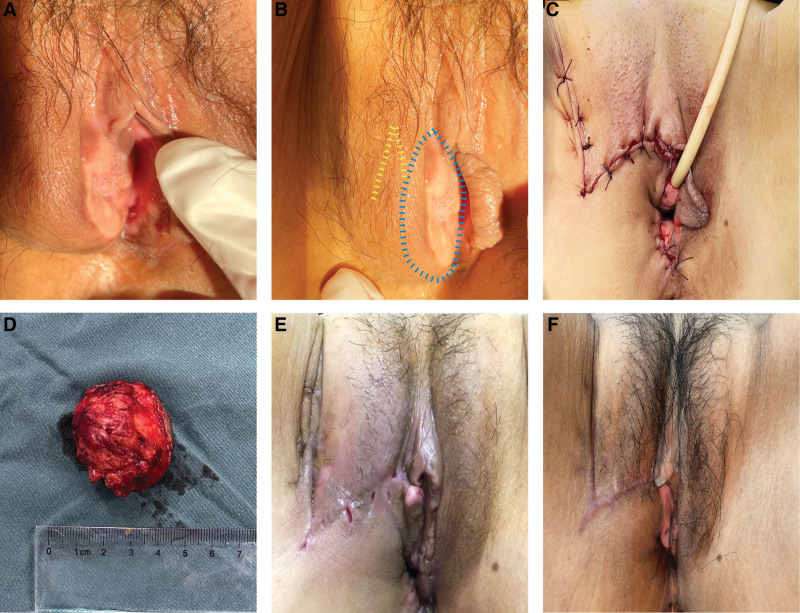
Clinical images. (A) A nodule in the right labium majus with a slightly granular surface was not adherent to the left labium majus as well as urethral orifice. (B) The blue dotted line, like an elliptical shape, showed the resection scope, and the yellow dotted line, like a Z-shape, showed the local flap for vulvar plasty. (C) The vulva after the closure of the surgical incision. (D) A tumor being excised showed unclear boundaries with surrounding tissues (only showing the base of the tumor). (E) Two-week postoperative follow-up image showed the site after removed stitches. (F) Two months postoperative follow-up image showed the surgical site healed completely.

A 47-years-old Chinese woman experienced a nodule on her right vulva with itch sometimes in late 2018. There was no pain, rupture, pus, and other symptoms at the local site. The patient reported it was small (about 0.5 × 0.5 cm) at onset and without receiving any treatment because the nodule did not bother her. On March 19, 2022, the patient complained the size of the nodule gradually increased with severe itch than before. Gynecologic examination noted an oval-shaped nodule of 3 × 2 cm with a slight ulcer surface on the right labium majus. It was skin colored, firm, tender, and unclear boundary with surrounding tissues (Fig. 1A). At the same time, there were several hard and palpable lymph nodes in bilateral groin. All above gave an impression of malignancy signs. Biopsy was performed under local anesthesia, and the pathology revealed GCT. In addition, MRI showed high possibility of vulvar cancer. We also used cystoscopy examination to investigate whether the tumor had bladder metastases, revealing no abnormality.

**Figure 2.**
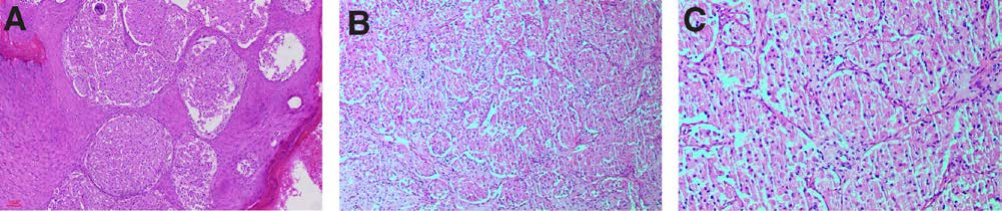
Histopathology features. (A) Initial biopsy of the tissue in Figure 1A (hematoxylin and eosin [H&E] stain, original magnification: A, ×100, B, ×200 and C, ×400) revealed the tumor cells located under the epidermis, and some tumor cell clusters were observed within the dermal papillae. A small amount of lymphocyte infiltration was found in the interstitium. The tumor cells were separated into nested clusters and sheets by fibrous capillaries. Furthermore, these cells exhibited abundant cytoplasm and a large number of eosinophilic granules with small nuclei, inconspicuous nucleoli, and rare nuclear division. Necrosis was absent.

**Figure 3.**
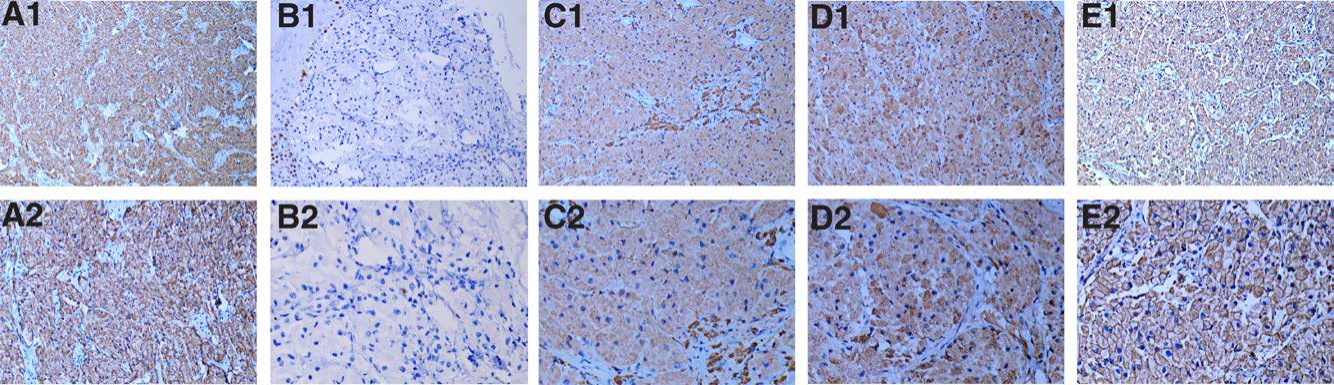
Histopathology of immunohistochemistry (IHC). All the specimens were from the initial biopsy tissue Figure 1A. A1 to E1 (original magnification ×200) were stained for CD56, Ki-67, CD68, S-100 and Vimentin, respectively. A2 to E2 (original magnification ×400) were also staining for CD56, Ki-67, CD68, S-100 and Vimentin respectively. In initial biopsy tissue, the tumor cells were strongly positive for CD56, CD68 and S-100, but a rare expression (1%) of Ki-67 was detected (brown color). IHC = immunohistochemical.

**Figure 4.**
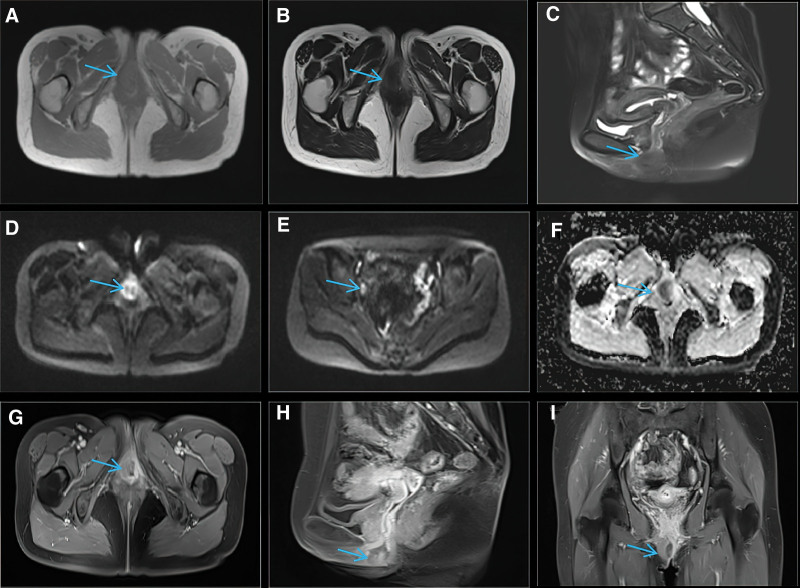
Magnetic resonance imaging (MRI). (A and B) The same transverse MR scan (*T*_1_-weighted and *T*_2_-weighted respectively), showed an irregular shape tumor on the right side of the vulva with isointensity signal, and the size of the nodule is 2.6 cm × 2.3 cm × 2.3 cm (blue arrow). (C) The fat-saturated median sagittal MR scan, showed mild hyperintensity signal (blue arrow). (D and E) The same transverse and diffusion-weighted imaging (DWI) MR scan respectively, showed hyperintensity signal and multiple lymph nodes in pelvic wall (blue arrow). (F) The transverse MR scan (apparent diffusion coefficient, ADC) map, showed hypointensity (blue arrow). (G to I) Transverse median sagittal and coronal contrast-enhanced MR scan respectively, showed uneven moderate enhancement (blue arrow). ADC = map hypointensity signal, DWI = diffusion-weighted imaging, MRI = magnetic resonance imaging.

Furthermore, Chest X-ray and abdominal sonography were unremarkable. Figure 2 shows histological presentation of abundant eosinophilic granular cytoplasm in the biopsy specimen of the nodule. Figure 3 reveals immunohistochemical stainings for marked protein CD56, Ki-67, CD68, S-100, Vimentin. The tumor cells demonstrate diffuse positivity for CD56, CD68, S-100, Vimentin, and a small amount of Ki-67 expression (1%) observed. Figure 4, the images of MRI show an irregular shape nodule on the right side of the vulva with *T*_1_ and *T*_2_ isointensity signal, diffusion-weighted imaging hyperintensity signal, apparent diffusion coefficient map hypointensity signal. The size of the nodule is 2.6 cm × 2.3 cm × 2.3 cm. In contrast-enhanced scan shows uneven moderate enhancement. Meanwhile, multiple lymph nodes are recorded in pelvic wall.

Based on the biopsy result before operation, the patient underwent local complete resection of vulvar tumor under general anesthesia on March 24, 2022. An elliptical incision was made on the skin around the vulva tumor. Then, a portion of vulvar skin and subcutaneous tissues were removed, and the resection scope was approximately 4 cm × 3 cm × 3 cm. Due to the large surgical incision, Z-plasty was performed to achieve the primary closure for decreasing wound tension and improving aesthetic reduction (Fig. 1B, C). Finally, a tumor that had a grayish red color, firm texture, and unclear boundary with surrounding tissues was totally cut off (Fig. 1D). The final pathological diagnosis was GCT of the vulva and surgical margins were uninvolved. With all these findings above, the diagnosis was “benign GCT.” At the 2-week post-surgery, stitches were removed and no swelling or split at the surgical site (Fig. 1E). At 2 months of postoperative follow-up, the surgical site healed completely (Fig. 1F).

## 3. Systematic review of the literature

This study was conducted on the Cochrane Collaboration and PRISMA statements. In this systematic literature search for GCT of the vulva, the database of PubMed, Cochrane Library, and Scopus (search date November 19, 2022) were searched by 2 independent reviewers (Guang-Yao Lin and Yan Liu) for eligible studies with the search terms: [All Fields] AND (“granular cell tumor” [All Fields] OR “granular cell tumor” [MeSH Terms] OR (“granular” [All Fields] AND “cell” [All Fields] AND “tumor” [All Fields]) OR “granular cell tumor” [All Fields]) AND (“vulva” [MeSH Terms] OR “vulva” [All Fields] OR “vulvas” [All Fields] OR “vulvae” [All Fields]).

In this review, 2 investigators (Tao Ye and Guang-Yao Lin) were screened for identified papers by inspecting appropriate titles and abstracts independently. The full-text of the relevant studies that reported GCT of the vulva were retrieved for data collection process. After a systematic screening independently, the following articles were excluded: Firstly, the cases were not confirmed as GCT of the vulva pathologically. Secondly, the literature could not be obtained or did not contain the full text. Thirdly, review, animal studies and non-English language articles. Baseline characteristics of all included studies were summarized by 1 author (Guang-Yao Lin) independently, according to a pre-designed standardized format including number of case, clinical presentation, time before diagnosis, tumor site, solitary or multiple of the tumor, immunohistochemistry results (positive), type of GCT, following time and prognosis.

## 4. Results

**Figure 5.**
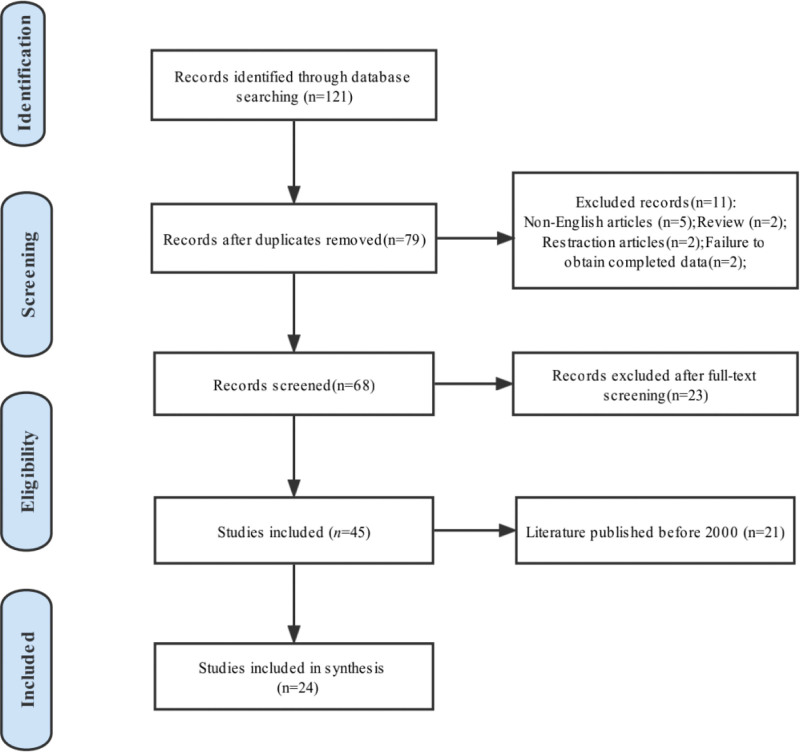
Paper selection flowchart.

After the selection procedure (Fig. 5), 79 potentially relevant studies were identified. Of those, 55 records were excluded because they were not published in English, and could not obtain full article or not reported on women with GCT of the vulva. Eventually, 48 citations were identified reporting on GCT of the vulva. As the previous review^[16]^ on this topic showed the available clinical information were limited in these literature published before 2000. Therefore, 24 studies published between the time interval of 2000 up to now were retrieved in full, and they were analyzed for this review. Table 1 shows the study characteristics of women with GCT of the vulva described in all 24 studies. In summary, 53 cases of GCT of the vulva have been reported in the literature.

**Table 1:** The summary of the literature describing women with GCT of the vulva.

Author	Yr	Number of case (n)	Clinical presentation	Time before diagnosis (m)	Tumor site	Solitary/Multiple	IHC (positive)	Type of GCT	Following time (m) and prognosis
Brunel^[17]^	2015	3	Asymptomatic/Pruritus/Pain	3/24/12	The pubis/Right labia majora × 2	Solitary × 3	S100 (diffuse expression)/N × 2	Atypical/N × 2	21/Recurrence 12/No recurrence 1/No recurrence
Vera-Sirera^[18]^	2014	1	Pain and pruritus	N	Left labium minus	Solitary	S100, Vimentin, CD57, Enolase, *α*-inhibin, CD68, Ki-67 (1%-2%)	Benign	15/No recurrence
Levavi^[19]^	2006	6	Pruritus/Enlarging mass × 2/Asymptomatic × 2/Palpable nodule	N	Right major labium × 3/Left major labium × 2/Multicentric vulva	Solitary × 5/Multiple	S100, Ki-67(<1%)	Benign	3 to 171/No recurrence
Zhang^[20]^	2011	1	Painless swelling	84	Both side of nympha and labia majora	Multiple	S100, NSE, calretinin, desmin	Benign	N
Kumarapeli^[21]^	2013	1	Painless nodule	1	Right labium	Solitary	S100, CD68, Ki-67 (10%–20%), p53 (10%-20%)	Atypical	24/No recurrence
Harou^[3]^	2015	1	Itchy	24	Left labium majus	Solitary	S100, NSE, NKI-C3, CD68, CD57, Calretinin, Ki-67	N	N
Kondi-Pafiti^[22]^	2010	4	Painless tumors	N	Left labia majora × 3/Right labia majora	Solitary × 4	S100, NSE	N	N/No recurrence
Althausen^[12]^	2000	13	Enlarging mass × 10/pain × 2/pruritis × 1	N	Labium majus × 8/The perianal area × 2/Introitus × 1/perineal body × 2	Solitary × 13	N	Benign	N
Ellison^[23]^	2003	1	Asymptomatic nodule	48	Left labia majus	Solitary	S100, D-PAS	Benign	N/No recurrence
Laxmisha^[24]^	2007	1	Asymptomatic lesion	12	Clitoris	Solitary	N	N	6/No recurrence
Cui^[4]^	2018	1	Asymptomatic lesion	12	Right labia majora	Solitary	S100, CD68	N	N/No recurrence
Garg^[25]^	2020	1	Growing swelling	48	Left labia majus	Solitary	S100, PAS	Benign	N
Baranova^[26]^	2021	1	Itching	N	Left labia and partially the left mons pubis	Solitary	S100, Ki-67 (1%–19%)	Synchronous malignant and benign	1/No recurrence
Cheewakriangkrai^[5]^	2005	1	Asymptomatic lesion	24	Left labia majus	Solitary	S100, NSE, PAS	N	N/No recurrence
Manning-Geist^[27]^	2017	4	Asymptomatic lesion × 2/Enlarged mass × 2	6/N/13/N	Right labia × 2/Right labia and mons/Left labia	Solitary × 3/Multiple	S100, Ki-67, calretinin, CD68,	Atypical × 1, benign × 3	0–144/No recurrence
Kavak^[28]^	2021	1	Enlarged mass	24	Left labia majus	Solitary	S100	N	N
Sonmez^[29]^	2016	1	Asymptomatic lesion	N	Right labia majus	Solitary	S100	Benign	7/No recurrence
Hong^[30]^	2013	4	Slow-growing lump/Labial cyst/Enlarged mass	108/12/N/12	Left labia majus/Right labia majus × 2	Solitary × 3	S100	Benign	N/16/97/15/No recurrence
Patabendige^[31]^	2019	1	Enlarged lump	24	Mons pubis	Solitary	S100	Benign	4/No recurrence
Rivlin^[13]^	2013	1	Growth lesion	144	Left labia majus	Solitary	S100	N	18/No recurrence
Kardhashi^[16]^	2010	2	Asymptomatic nodular × 2	72/24	Left labia majus × 2	Solitary × 2	S100, CEA	N	72/No recurrence 10/Recurrence
Ramos^[7]^	2000	1	Enlarged mass	8	Left labia majus	Solitary	S100, CD68, Ki-67 (5%–10%)	Malignant	16/No recurrence
Schmidt^[14]^	2003	1	N	N	Left labia majus spreading to the mons pubis	Solitary	S100, p53 (10%), MIB-1 (20%)	Malignant	14/Pulmonary,hepatic and skeletal metastases
Mehta^[32]^	2010	1	Increased lesion and new lesion, itching	480	Right labia majus	Multiple	S100, PAS	N	N

GCT = granular cell tumor, IHC = immunohistochemical, N = not mention.

**Figure 6.**
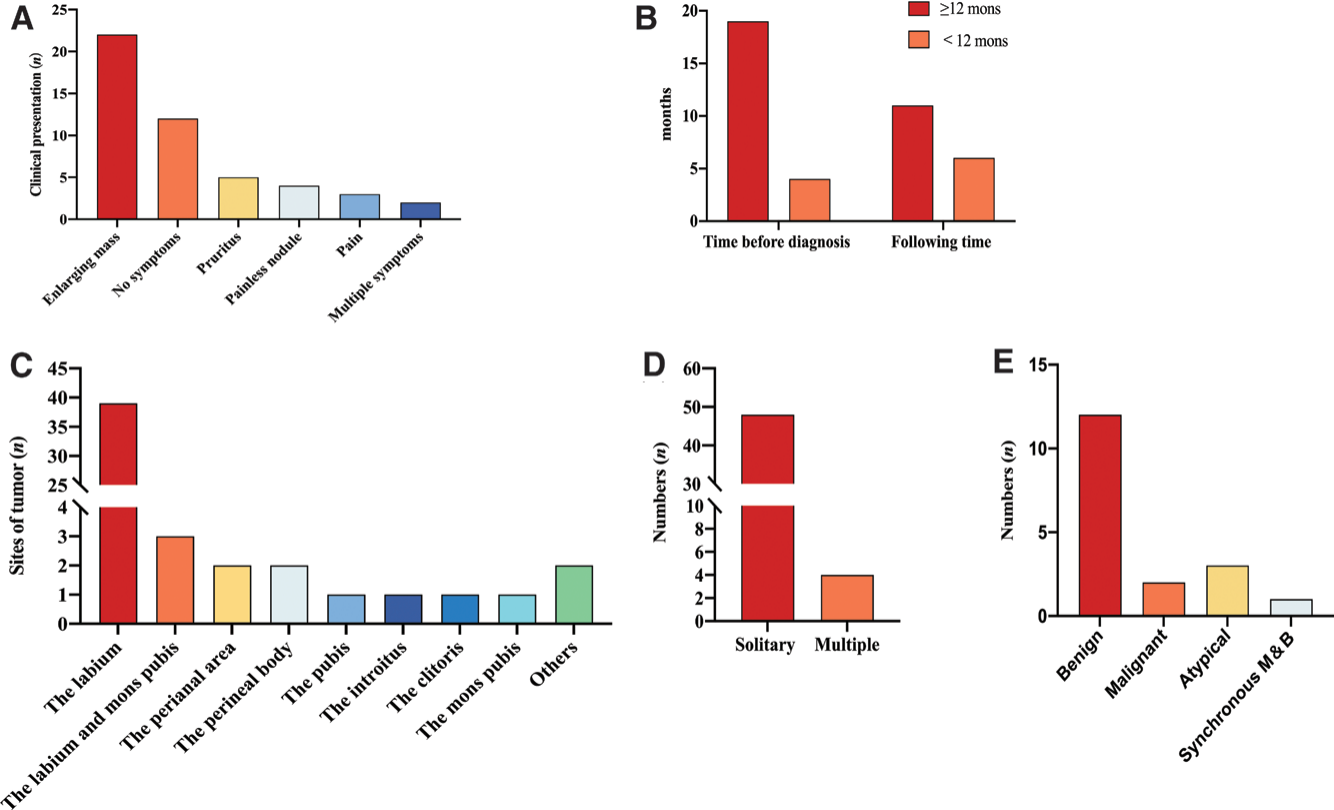
The summary of the literature describing women with GCT of the vulva. GCT = granular cell tumor.

At the time before histologic diagnosis, clinical presentation, sites of tumor, solitary or multiple local lesions, type of GCT (benign, atypical, malignant), the positive result of immunohistochemistry, following time and prognosis were recorded. The time between initial symptoms emerged and got histologic diagnosis was from 1 to 480 months. Except for 2 studies,^[12,24]^ 22 studies report S100 was positive for immunohistochemistry. Figure 6A shows the most common clinical presentation of GCT of the vulva was enlarged mass reported in 22 cases and following no symptom reported in 12 cases. Figure 6B shows diagnostic delay was often over 12 months in 19 cases reported in this pooled analysis. On the following time of GCT of the vulva, however, beyond 12 months was reported in 11 cases and less than 12 months reported in 6 cases which accounts for half of the former. There is no unique location for GCT of the vulva, as Figure 6C reveals, but the labium was the most commonly presented site. In addition, the solitary local lesion was reported in 48/53 (91%) of cases (Fig. 6D). 14 studies described the type of GCT of the vulva clearly, including 18 benign cases, 2 malignant cases, 3 atypical cases, and 1 synchronous malignant and benign case (Fig. 6E). Overall, women with GCT of the vulva is rare and is difficult to be diagnosed correctly in time for its diverse clinical presentation.

The largest patient study of women with GCT of the vulva included in this systematic review was published by Althausen AM et al^[12]^ The authors described 13 women (mean age 52) with GCT of the vulva treated at the Hartford Hospital between April 1982 and May 1998. In this study, the diagnosis for GCT of the vulva was defined through pathology specimen. Almost all women presented initially symptomatic solitary lesions. Tumor size was available for 12 cases and median size was 1.1 cm at excision (range from 0.3 to 2.3 cm). Follow-up data showed 5 cases with confluent expansile margin had been no recurrences. In the remaining 8 cases with irregular and infiltrative margin, 5 of the 8 patients experienced recurrences, despite 3 of the 5 cases had negative surgical margins. Therefore, infiltrative and ill-defined surgical margins at resection were the most important prognostic factor regarding recurrence.

Three studies^[7,14,26]^ reported 2 cases of malignant as well as 1 synchronous malignant and benign GCT of the vulva were identified. Firstly, Ramos et al^[7]^ reported a 17 years patient with malignant GCT of the left labia majus, of whom were treated with left hemivulvectomy and left groin lymph node dissection soon after local resection, which involved positive surgical resection margin. The girl received postoperative radiation therapy with a total dose of 60.8 Gy, and there was no sign of recurrence after a follow-up of 16 months. Furthermore, Schmidt et al^[14]^ reported another malignant case with the tumor site in left labia majus spreading to the mons pubis. Partial resection of the vulva was carried, although surgical resection margins were reached with GCT and postoperative therapy lacked. Two years later, she experienced femoral and extraperitoneal pelvic lymphadenectomy for several new recurrent tumors in the vulvar region. Unfortunately, the patient reported pulmonary, hepatic and skeletal metastases and died of hepatic failure 4 months after demission. Interestingly, Baranova et al^[26]^ presented a 50 years old postmenopausal patient with 7 × 4 cm tumor in the left labia and partially the left mons pubis. The patient initially underwent biopsy firstly and the histological results demonstrated an infiltrative lesion with positive for S100 protein immunohistochemically. Then a total deep vulvectomy and a left-sided inguinal femoral lymphadenectomy were performed. The larger and smaller lesions were histologically malignancy with pelvic lymph nodes metastases and benign, respectively. Both lesions were noted with a negative resection margin. And short follow-up time (1 month) did not show local surgical complications. A conclusion based on the malignant cases above reveals that, after excluding distant metastases, radical surgery with negative resection margins and postoperative radiation therapy might improve the prognosis.

The bulk of studies included in our systematic review were small case reports. However, as shown in Figure 6, most the presenting complaints of GCT of the vulva were solitary enlarged mass in the labium. Although most of GCT of the vulva were benign, they were not histologically diagnosed promptly and did not get long-term following time after completed tumor resection.

## 5. Discussion

In this case, report combined with systematic review of the literature, a conclusion we draw is that GCT of the vulva is a rare disease and surgery is the mainstay of therapy strategy for localized disease while radiotherapy may be appropriate for women with malignant GCT of the vulva. Specifically, we identified and analyzed 24 studies with 53 cases of GCT of the vulva, and diagnostic delay between initial clinical presentation to histological diagnosis was often over 12 months which might be caused by the reason that it is difficult to distinguish from sebaceous cysts, lipomas, fibromas, angiosarcoma, hidradenomas, papillomas, Bartholin duct cyst, leiomyoma, an epidermal cyst. Overall, the prognosis of a patient with benign GCT of the vulva is acceptable, but long-term following time is necessary.

The majority of GCTs are benign, although approximately 1% to 2% of cases may be malignant, which has a high rate of metastases as well as a short survival.^[8,33]^ In daily practice, Fanburg-Smith criteria were often adopted to classify GCT into benign, atypical, and malignant types. Whereas these criteria are subject to interobserver variation and the reproducibility among different pathologists is weak as well. Therefore, to form a simpler and more practical and distinct diagnostic criteria, Nasser et al^[9]^ in 2011 compared their reclassification results using only necrosis and/or mitoses, based on 48 cases with GCT, with results according to Fanburg-Smith study. Of note, their results demonstrated similar selectivity of 2 more reproducible criteria and metastases remain the unique explicit criterion for malignancy. In addition, Ki-67 immunostain values greater than 10% can help to classify malignant cases histologically.^[8,34]^ Before planning treatment, we should exclude multicentric lesions via taking a detailed history combined with systematic physical examination and exclude metastases by imaging examinations, like chest X-ray, abdominal sonography or pelvic MRI. Due to the rarity of the disease, there is clearly, no standard surgery regimen for GCT of the vulva. While since this disease commonly has no solid lesions and neoplasm cells may infiltrate local tissues. Cui et al^[4]^ suggested the extent of surgery excision could be extended to a region without infiltration to ensure a clear margin, based on the full resection of the lesion. Recurrence rates were reported as 20% with positive margins at resection and, by comparison, 2% to 8% with clear margins.^[13]^ Therefore an appropriate management approach may be to propose re-excision of the “infiltrative margins” nodules rather than clinical observation alone.^[12]^ We recommend that it is advisable to identify classification of the GCT before plan treatment through a biopsy or to have margins evaluated intraoperatively by frozen section, clearly, such that extended local excision can be achieved for positive margins while operating. Malignant GCT is a high grade-invasive disease with widespread metastases 3 to 5 years after diagnosis, which means lymphadenectomy and vulvectomy were needed to improve the prognosis.^[7,26,33]^ Whether adjuvant chemotherapy or radiotherapy for malignant GCT remains controversial. For example, Leib et al^[35]^ disapproved radiation therapy was used for GCT of the vulva. Due to its meager vascularity, the neoplasm tolerates vascular reduction stronger than the surrounding tissue. Others used^[14]^ postoperative radiation therapy with a total dosage of 60.8 Gy and no recurrence after 16 months of follow-up.

It is of note that the treatment strategies for surgical wound, especially for large tumors in the vulvar region, is also another challenge for gynecologists. Though there are tremendous different techniques for labiaplasty, the essential goals when performing a labiaplasty should include maintenance of the neurovascular supply, minimal invasiveness, and no impairment of sexual dysfunction.^[36]^ Z-plasty is a popular plastic surgical procedure applied for relieving of cicatricial contraction and reducing tension. As early as in 1963, Scott et al^[37]^ described the advantages and key procedures of Z-plasty adopted for vulvectomy, including eliminating the circular type scar as well as incapacitating constrictor. In this case, we performed an additional Z-shaped incision in the opposite direction at the side of the axial incision as showed in Figure 1B above. After the subcutaneous skin was fully free, 2 diagonal triangular flaps were formed, and the positions were exchanged and sutured with each other. This is the first case, to our knowledge, reported the Z-plasty for GCT of the vulva. At 2-month follow up, there was no complications at the surgical site and no perineal discomfort. The outcomes in this case verify that Z-plasty is a valuable and simple technique for GCT of the vulva in the labium, even with large vulvar defects after resection. However, future studies should verify this approach.

The case report demonstrated within this review had typical features of GCT of the vulva. For example, GCT of the vulva usually appears on the labium but also in other location such as the labium and mons pubis, perianal and perineal. Since many of these lesions were described as enlarged mass or no symptom, they were often diagnosed as an incidental finding or during an unrelated procedure. We chose local extended resection with negative margins as the primary therapy derived from our consultation of the literature. We chose local complete excision with negative margins as the primary therapy derived from our consultation of the literature. For example, almost every patients with benign CGT of the vulva, in our literature review, underwent local excision with wide margins. If resection margins are involved, extended local excision may be involved. In addition, the patient was diagnosed as benign GCT, because there was no malignant signs like cellular atypia, tumor necrosis, increasing mitotic activity, large nucleoli, high nuclear and prominent spindling. Also, all tumors stained strongly positive for the CD56, CD68 and S-100 protein, but immunostaining for Ki-67 was rare, positive in less than 1%. Clearly, a lymph node biopsy could have been performed in this patient before operation, because there were several hard and palpable lymph nodes in bilateral groin. However, she declined and lymph nodes in bilateral groin could not be detected as well as no new lesions were found after 8 months follow-up.

## 6. Conclusion

In conclusion, we found that GCT of the vulva is rare, and the most common location is in the labium with the presenting complaints of enlarging mass. The prognosis of women with benign GCT of the vulva is acceptable. Surgery with negative resection margins is the mainstay of treatment, while Z-plasty is appropriate for decreasing the tension of the wound and improving aesthetic reduction.

## Author contributions

**Conceptualization:** Guang-Yao Lin, Tao Ye.

**Data curation:** Tao Ye.

**Formal analysis:** Tao Ye.

**Funding acquisition:** Tao Ye.

**Investigation:** Yan Liu, Tao Ye.

**Methodology:** Yan Liu, Xin-Yu Lu.

**Project administration:** Xin-Yu Lu.

**Resources:** Guang-Yao Lin, Yan Liu.

**Software:** Jie Gao.

**Supervision:** Xin-Yu Lu, Jie Gao, Yong-Zhou Wang.

**Validation:** Xin-Yu Lu.

**Visualization:** Jie Gao.

**Writing – original draft:** Guang-Yao Lin.

**Writing – review & editing:** Yong-Zhou Wang.
